# BCL6B-dependent suppression of ETV2 hampers endothelial cell differentiation

**DOI:** 10.1186/s13287-024-03832-y

**Published:** 2024-07-29

**Authors:** Zhonghao Li, Wei Wu, Qiushi Li, Xin Heng, Wei Zhang, Yinghong Zhu, Lin Chen, Ziqi Chen, Mengcheng Shen, Ning Ma, Qingzhong Xiao, Yi Yan

**Affiliations:** 1 Department of Cardiology, Translational Research Center for Regenerative Medicine and 3D Printing Technologies, Guangdong Provincial Key Laboratory of Major Obstetric Diseases; Guangdong Provincial Clinical Research Center for Obstetrics and Gynecology, The Third Affiliated Hospital, Guangzhou Medical University, Guangzhou, 510150 China; 2 Department of Pathophysiology, School of Basic Medical Sciences, Southern Medical University, Guangzhou, 510515 China; 3 Stanford Cardiovascular Institute, Stanford University School of Medicine, Stanford, 94305 USA; 4 Guangzhou National Laboratory, Guangzhou, 510005 China; 5 Centre for Clinical Pharmacology and Precision Medicine, William Harvey Research Institute, Faculty of Medicine and Dentistry, Queen Mary University of London, London, EC1M 6BQ UK; 6 Key Laboratory of Cardiovascular Diseases at The Second Affiliated Hospital of Guangzhou Medical University and Guangzhou Municipal, Guangdong Provincial Key Laboratory of Protein Modification and Degradation, School of Basic Medical Sciences, Guangzhou Medical University, Guangzhou, 511436 China

**Keywords:** B-cell CLL/lymphoma 6 member B, Human induced pluripotent stem cell, Endothelial cell, Vessel organoids, ETS variant transcription factor 2

## Abstract

**Background:**

B-cell CLL/lymphoma 6 member B (BCL6B) operates as a sequence-specific transcriptional repressor within the nucleus, playing crucial roles in various biological functions, including tumor suppression, immune response, stem cell self-renew, and vascular angiogenesis. However, whether BCL6B is involved in endothelial cell (EC) development has remained largely unknown. ETS variant transcription factor 2 (ETV2) is well known to facilitate EC differentiation. This study aims to determine the important role of BCL6B in EC differentiation and its potential mechanisms.

**Methods:**

Doxycycline-inducible human induced pluripotent stem cell (hiPSC) lines with BCL6B overexpression or BCL6B knockdown were established and subjected to differentiate into ECs and vessel organoids (VOs). RNA sequencing analysis was performed to identify potential signal pathways regulated by BCL6B during EC differentiation from hiPSCs. Quantitative real-time PCR (qRT-PCR) was used to detect the expression of pluripotency and vascular-specific marker genes expression. EC differentiation efficiency was determined by Flow cytometry analysis. The performance of EC was evaluated by in vitro Tube formation assay. The protein expression and the vessel-like structures were assessed using immunofluorescence analysis or western blot. Luciferase reporter gene assay and chromatin immunoprecipitation (ChIP)-PCR analysis were used to determine the regulatory relationship between BCL6B and ETV2.

**Results:**

Functional ECs and VOs were successfully generated from hiPSCs. Notably, overexpression of BCL6B suppressed while knockdown of BCL6B improved EC differentiation from hiPSCs. Additionally, the overexpression of BCL6B attenuated the capacity of derived hiPSC-ECs to form a tubular structure. Furthermore, compared to the control VOs, BCL6B overexpression repressed the growth of VOs, whereas BCL6B knockdown had little effect on the size of VOs. RNA sequencing analysis confirmed that our differentiation protocol induced landscape changes for cell/tissue/system developmental process, particularly vascular development and tube morphogenesis, which were significantly modulated by BCL6B. Subsequent experiments confirmed the inhibitory effect of BCL6B is facilitated by the binding of BCL6B to the promoter region of ETV2, led to the suppression of ETV2's transcriptional activity. Importantly, the inhibitory effect of BCL6B overexpression on EC differentiation from hiPSCs could be rescued by ETV2 overexpression.

**Conclusions:**

BCL6B inhibits EC differentiation and hinders VO development by repressing the transcriptional activity of ETV2.

**Supplementary Information:**

The online version contains supplementary material available at 10.1186/s13287-024-03832-y.

## Background

B-cell CLL/lymphoma 6 member B (BCL6B), also referred to as BAZF, ZNF62 and ZBTB28, belongs to the B-cell lymphoma 6 (BCL6) gene family [1]. Members of this family encode zinc finger proteins with the Broad-Complex, Tramtrack and Bric a brac/POxvirus and Zinc finger (BTB/POZ) domain, functioning as sequence-specific transcriptional repressors. The zinc finger motifs and BTB/POZ domain of BCL6B are 94% and 65% identical to those of BCL6 at the amino acid level [1]. Although binding to similar target DNA sequences to act as transcriptional repressors, the tissue expression pattern and pathological function of BCL6B differ from that of BCL6. Some studies have indicated that BCL6B is involved in diverse biological functions, such as facilitating spermatogonial stem cell self-renewal [2], stimulating secondary response of memory CD8 + T lymphocytes [3], and repressing hepatocellular carcinoma [4, 5], colorectal carcinoma [6, 7], and breast cancer [8]. A recent study by Miruto Tanaka et al. showed that BCL6B contributes to ocular vascular diseases via Notch signal silencing [9]. BCL6B is enriched in endothelial cells (ECs) [10], however, as a transcriptional repressor, the function and mechanisms through which BCL6B regulates EC development remain poorly understood.

The two-dimensional (2D) cell culture system has been successfully applied in biomedical fields over the past 100 years which has significantly help us to improve our knowledge of cellular signaling pathways, develop guidelines for the design of candidate compounds, identify potential drug targets, and clarify the underlying pathological mechanisms of diseases. However, such a system comes with many disadvantages such as lack of hierarchical structure, dimensionality, cellular diversity, and cell–cell or cell–matrix interactions, all of these properties are crucial for maintaining the in vivo cellular functions and tissues homeostasis. Such a limitation can be circumvented by the newly established human organoids. 3D human organoids derived from primary tissues or stem cells can self-renew, and self-organize, and exhibit multicellularity and functionality similar to those of in vivo organs. Particularly, human induced pluripotent stem cells (hiPSCs) have the capability to differentiate into various cell types [11], which allows for studying human development, cell differentiation and the role of critical transcriptional factors in cell fate decision [12]. Importantly, recent advances in hiPSC-derived organoids enable a better recapitulation of the three dimensional (3D) microstructures and functions of human tissues [13]. HiPSC-derived organoids provide a more sophisticated tool and model system for exploring the critical signal pathways underlying human embryonic development and cell differentiation, modeling human diseases, uncovering disease aetiology, and screening drug candidates [14].

ETS variant transcription factor 2 (ETV2), also known as ER71, a member of the E26 transformation-specific transcription factor family, has been identified as a master transcription factor for the hematoendothelial lineage development [15, 16]. ETV2 overexpression in mouse embryonic stem cells directly regulates Flk1 expression and initiates the de novo generation of ECs [17, 18], and ETV2 alone is sufficient to directly convert human fibroblasts into ECs [19, 20]. Although much effort has been made to study the importance of ETV2 in EC differentiation, little is known about its upstream regulators. In zebrafish, transcription factor FOXC1a/b serves as a direct upstream regulator of Etsrp (orthologous to human ETV2) and involves in mesoderm specification into angioblasts [21]. However, the upstream regulators of ETV2 in mammal still remains unclear.

In this study, utilizing both hiPSC-derived 2D ECs and 3D vessel organoids (VOs), we uncovered that BCL6B hampers EC differentiation and VO development from hiPSCs by repressing the transcriptional activity of ETV2.

## Methods

### Cell culture and EC differentiation

hiPSCs (passage 25–40) were maintained under feeder-free conditions in defined E8 media (Gibco, A1516901) on tissue culture plates coated with hESC-qualified Matrigel (Corning, 354277) at 37 °C in 5% CO2. The culture medium was exchanged daily. Routine passaging of hiPSCs at 1:4, and single-cell dissociation were carried out using Accutase solution (Sigma, A6964). The hiPSCs were cryopreservation with CryStor CS10 (Stemcell, 100–1061). In addition, we performed a mycoplasma contamination test every two weeks to ensure the health of all the cell lines (data not shown).

The EC differentiation protocol was adapted from a published protocol [22]. In brief, hiPSCs were cultured in Essential 8 medium reached ~ 80% confluency, then were dissociated into single cells, and seeded in 6-well plates with 10 μM ROCK inhibitor Y27632 (Selleck, S1049) on D0 at a density of 10^5^ cells/well. On D1, hiPSCs were treated with 8 μM CHIR99021 (Selleck, S2924) in Chemically Defined Medium (CDM) for 3 days. On D4, differentiated cells were treated with 50 ng/ml VEGF (PeproTech, 100–20) and 10 mM SB431542 (Selleck, S1067) until cells reached ~ 100% confluent on D6. For cell dissociation, TrypLE Select Enzyme (10 ×) (Gibco, A1217701) was used for 10 min until most cells became singlets. The cells were then passed through a 40-μm strainer. Cell dissociation was neutralized by adding 2 ml of Endothelial Cell Medium (ECM, ScienCell, 1001), followed by magnetic associated cell sorting (MACS) to purify hiPSC-derived ECs with CD144 (VE-Cadherin) MicroBeads (Miltenyi, 130-097-857) or subjected to flow cytometry analysis. The CDM consists of IMDM (50%); Ham's F12 Nutrient Mix (50%); BSA (0.25%); Lipid concentrate (1X); ITS (0.1%); Ascorbic acid (50 µg/ml); Monothioglycerol (450 µM); and Glutamax (1X).

Human umbilical vein endothelial cells (HUVECs) were purchased from OriCell® (Cat. HUVEC-20001), and cultured in Endothelial Cell Medium (ECM; ScienCell, 1001) supplemented with 5% heat-inactivated fetal bovine serum (ScienCell, 0025), and 1% endothelial cell growth supplement (ECGS; ScienCell, 1052). All HUVECs in this paper were used by passage 5. HEK293T cells were maintained in our lab with DMEM medium (Gibco, 11965092) supplemented with 10% fetal bovine serum (PAN, ST30-3302). All cells were cultured at 37 °C and 5% CO_2_.

### Generation of human blood VOs

A step-by-step protocol detailing the differentiation of the human VOs can be found in Nature Protocol [23]. Briefly, hiPSCs were dissociated into single cells with Accutase. Subsequently, the cells were resuspended in the E8 medium containing 10 μM Y27632 and seeded at 1000 cells/well in round bottom ultra-low attachment 96-well plates (Costar, 7007) on D-1, with a volume of 100 μl per well. The plate was then centrifuged at 100 g for 3 min to assemble hiPSCs into embryoid bodies (EBs) and placed in an incubator at 37 °C with 5% CO2. After 24 h (D0), the culture medium was replaced by the N2B27 medium with 12 μM CHIR99021 and 30 ng/ml BMP4 (PeproTech, 120–05). On D3, the N2B27 medium was renewed with 100 ng/ml VEGF and 2 μM forskolin (HY-15371). Starting from D6, the resulting cell aggregates were embedded in Matrigel:Collagen I (1:1) gels and overlaid with ECM containing 15% FBS, 100 ng/mL VEGF and 100 ng/ml FGF-2 (PeproTech, 100-18B). This medium was changed every two days. Around D10-12 vascular networks were established, and either directly analyzed or networks from individual cell aggregates were extracted from gels and further cultured in round bottom ultra-low attachment 96-well plates. These vascular networks self-assembled into vascular organoids, and the medium was changed every three days until organoids were ready for analysis.

### Establishment of BCL6B overexpression and knockdown hiPSC lines

The human BCL6B coding regions, fused with Flag tags, was amplified through polymerase chain reaction (PCR) using hiPSC cDNA as template. Subsequently, these templates were cloned into adapted FUW-tetO-MCS vector (MiaoLingBio, P48786). Concurrently, shRNA constructs were generated using the Tet-pLKO-puro vector (MiaoLingBio, P0171), following previously described methods [24]. For viral production, 10 µg of target vectors (BCL6B-FLAG, BCL6B shRNA, and FUW-M2rtTA (MiaoLingBio, P0521), along with lentiviral envelope and the packaging plasmids psPAX2 (MiaoLingBio, P0261) and pMD2.G (MiaoLingBio, P0262), were transfected into 293 T cells using lipofectamine 3000 (Invitrogen, L3000015). After 72 h, lentivirus supernatants were collected and filtered through a 0.45 µm syringe filter. hiPSCs were coinfected with BCL6B-FLAG and FUW-M2rtTA or infected solely with BCL6B shRNA lentivirus using a spin infection method, as previously described [25]. Two days post-infection, puromycin-resistant clones positive clones were selected using 1 μg/ml puromycin (MCE, HY-B1743A) for 4 days to establish BCL6B overexpression (BCL6B OE) or BCL6B knockdown (BCL6B KD) hiPSC lines, respectively. The successful establishment of hiPSC lines was confirmed by western blotting and then applied for later differentiation.

### Immunofluorescence analysis

For hiPSCs-derived ECs, the purified ECs were seeded in a 24-well plate with coverslips and cultured until reaching 100% confluence. The cells were fixed in 4% paraformaldehyde (PFA) for 15 min, permeabilizated with 0.5% Triton X-100 for 10 min, followed by blocking with 10% goat serum for 30 min. Subsequently, the cells were incubated with mouse anti-CD31 (Abcam, ab9498) and rabbit anti-VE Cadherin (ab33168) at 4 °C overnight. Secondary antibodies used were Alexa Fluor 488-conjugated goat anti-rabbit (Abcam, ab150077) and Alexa Fluor 647-conjugated goat anti-Mouse (Abcam, ab150115). DAPI (Sigma, D9542) was used to mark cell nuclei. Stained coverslips were mounted with mounting medium and stored at 4 °C before imaging. All images were acquired by confocal imaging systems.

For whole-mount staining of organoids, the samples were fixed in 4% PFA for 1 h at room temperature (RT), followed by three washes with PBS. Subsequently, the organoids were incubated in 0.5% TritonX-100 at RT for 1 h. After blocking with 5% BSA in 0.1% TritonX-100 at RT for 1 h, organoids were incubated with primary antibodies at 4 °C for 48 h, washed with PBS, and then incubated with secondary antibodies at 4 °C for 48 h. The stained organoids underwent three washes with PBS before being mounted on glass microscope slides using Mounting Medium (Invitrogen, P36961). To preserve the 3D structure of the organoids before confocal imaging, Polybead Microspheres were placed between the slide and the coverslip.

### Flow cytometry analysis

HiPSC-ECs (D6) were dissociated into single-cell suspensions using TrypLE Select Enzyme for 8–10 min. After resuspension in staining buffer (Biolegend, 420201), approximately 0.5 × 10^6^ single cells for each group were incubated with PE anti-human CD144 antibody (Biolegend, 348506) and FITC anti-human CD31 antibody (Biolegend, 303104) for 30 min. The results were analyzed using the BD LSR FortessaX-20 cytometer and FlowJo software.

The vascular organoids (D15) were mechanically disrupted and disaggregated using 3U/mL Dispase (Gibco, 17105041), 2U/mL Liberase (Sigma, 5401020001) and 100U DNAse (Stemcell, 07900) in PBS for 30 min at 37 °C while rotating. Subsequently, single cells were stained with the following antibodies for 30 min: PE anti-human CD144 antibody (Biolegend, 348506), FITC anti-human CD31 antibody (Biolegend, 303104) and APC anti-human PDGFRβ antibody (Biolegend, 323608), followed by FACS analysis.

### RNA extraction, cDNA synthesis, and qRT-PCR

The total RNA of hiPSCs, hiPSC-ECs and 10 VOs were extracted using Trizol (Invitrogen, 15596026), followed by reverse transcription to generate cDNA using the HiScript II 1st Strand cDNA Synthesis Kit (Vazyme, R212-01). qRT-PCR was performed using the Applied Biosystems QuantStudio 5 qPCR system with the ChamQ SYBR qPCR Master Mix (Low ROX Premixed) (Vazyme, Q331-02). Relative mRNA expression was determined by the delta cycle time with human GAPDH serving as the internal control for data normalization. The primer sequences were as follows:

GAPDH: forward, 5′-TCGGAGTCAACGGATTTGGT-3′, reverse, 5′-TTCCCGTTCTCAGCCTTGAC-3′;

NANOG: forward, 5′-CAATGGTGTGACGCAGAAGG-3′, reverse, 5′-TGCACCAGGTCTGAGTGTTC-3′;

OCT4: forward, 5′-CTCGAGAAGGATGTGGTCCG-3′, reverse, 5′-TGACGGAGACAGGGGGAAAG-3′;

PECAM1: forward, 5′-AGACGTGCAGTACACGGAAG-3′, reverse, 5′-TTTCCACGGCATCAGGGAC-3′;

VE-Cadherin: forward, 5′-CGCAATAGACAAGGACATAACAC-3′, reverse, 5′-GGTCAAACTGCCCATACTTG-3′;

VWF: forward, 5′-CCCGAAAGGCCAGGTGTA-3′, reverse, 5′-AGCAAGCTTCCGGGGACT-3′;

VEGFR2: forward, 5′-GAGGGGAACTGAAGACAGGC-3′, reverse, 5′-GGCCAAGAGGCTTACCTAGC-3′;

PDGFRβ: forward, 5′-ATCAGCAGCAAGGCGAGC-3′, reverse, 5′-CAGGTCAGAACGAAGGTGCT-3′.

### Western blotting

Cell pellets were lysed using RIPA buffer containing 1% PMSF. Protein concentration was measure by the BCA method using a Pierce BCA Protein Assay Kit (23225, Thermo Fisher Scientific). Samples were resolved on 10% sodium dodecyl sulfate polyacrylamide gels (SDS-PAGE), followed by transfer to a PVDF membrane at 100v for 120 min. Membranes were blocked with 5% non-fat dry milk in 1xTBST at RT for 1 h and then incubated with primary antibody at 4 °C overnight. The following antibodies were used: mouse anti-CD31 (Abcam, ab9498); rabbit anti-VE Cadherin (ab33168); rabbit anti-VWF (ab6994); rabbit anti-BCL6B (Origene, TA369826); rabbit anti-FLAG (Abcam, ab205606); and mouse anti-beta actin (Abcam, ab8226). After appropriate washing with 1xTBST, the membranes were incubated with Goat Anti-Rabbit IgG H&L (HRP) (Abcam, ab6721) or Goat Anti-Mouse IgG H&L (HRP) (Abcam, ab6789) at RT for 1 h. A chemiluminescent assay was performed using ECL substrates (Abcam, ab133406), and signals were detected with a FUSION Solo S chemiluminescence imaging system (5200 Muti, Tanon).

### Tube formation assay

hiPSC-ECs were seeded onto 96-well plates coated with 50 µl matrigel (BD, 356231) at a density of 2 × 10^4^ cells per well and then incubated for 4 h. Tubular structures were photographed under a light microscope (Zesis) and analyzed using Image J software (NIH, Bethesda, MD).

### Luciferase reporter assay

Luciferase reporter transfection and dual-luciferase assays were performed as follows. Briefly, HEK293T cells were seeded at 5 × 10^4^ cells/well in 24-well plates and transfected using Lipofectamine 2000 (Invitrogen, 11668019) with 0.8 μg reporter vector carrying firefly luciferase (Promega, E1751) and the indicated target sequences. The PRL-TK vector (carrying Renilla luciferase; Promega, E2241) was co-transfected as an internal control. At 24 h after transfection, cells were lysed using lysis buffer (Vazyme, DL101-01) and subjected to a dual-luciferase assay using the Dual-Luciferase® Reporter (DLR™) Assay System (Promega, E1910) according to the manufacturer’s protocol.

### RNA sequencing (RNA-Seq) analysis

A total amount of 1 µg RNA was used for the RNA sample preparations. Sequencing libraries were generated using NEB-Next® Ultra™ RNA Library Prep Kit for Illumina® (NEB, USA). The clustering of the index-coded samples was performed on a cBot Cluster Generation System using TruSeq PE Cluster Kit v3-cBot-HS. After cluster generation, the libraries were sequenced on an Illumina Novaseq platform. Differential expression analysis of two conditions/groups (three biological replicates per condition) was performed using the R package DESeq2 (1.16.1). Genes with an adjusted *P*-value < 0.05 found by DESeq2 were assigned as differentially expressed. Gene Ontology (GO) enrichment analysis of differentially expressed genes (DEGs) was applied using the R package clusterProfiler. GO terms of DEGs with corrected P value less than 0.05 were significantly enriched. We used the R package clusterProfiler to assess the enrichment of DEGs in Kyoto Encyclopedia of Genes and Genomes (KEGG) pathways. The RNA sequencing data in this study were archived in supplementary table.

### Chromatin immunoprecipitation (ChIP) assay

After the cells were cross-linked, the ChIP assay was performed using a ChIP kit (CST, 9003S). Specifically, for each immunoprecipitation reaction, the nuclear fraction from 2 × 10^7^ cells and 3 μg of HA-tag antibody (CST, 3742S) or control IgG were used. Purified DNA fragments were utilized for subsequent PCR amplification with the following specific primer sets: forward, 5′-GCAAGTGGATCACTTGAGGT-3′, reverse, 5′-TGCGATCTCAGCTCACTGCAA-3′. The resultant PCR products (expected amplified size is 190 bp) were analyzed by performing 2% (w/v) agarose gel electrophoresis.

### Statistical analysis

Results are presented as mean ± SD from a minimum of three independent experiments. Group comparisons were conducted using the non-paired two-tailed Student's t-test. In cases of non-normal distribution, the two-tailed F test was employed. For comparisons involving more than two groups, ANOVA was applied. A *p* value of < 0.05 were considered statistically significant.

## Results

### Generation and characterization of ECs from hiPSCs

To investigate whether BCL6B plays a functional role in endothelial cell (EC) development, a feeder-free, and chemically defined protocol (Supplementary figure: Fig. S1A) was adapted in this study to generate ECs from human induced pluripotent stem cells (hiPSCs). Throughout the differentiation process, cell morphology was gradually changed (Supplementary figure: Fig. S1B). Differentiated hiPSC-derived ECs were purified using a CD144 antibody before being re-plated for further characterization on D7 (Supplementary figure: Fig. S1C). The purified cells displayed a typical EC morphology (Supplementary figure: Fig. S1C) and were positive for canonical EC markers, VE-Cadherin and CD31 (Supplementary figure: Fig. S1C). We further confirmed that hiPSC-derived ECs shared a comparable capillary-like tube formation capacity with HUVECs (Supplementary figure: Fig. S1D). Quantitative data revealed similar junction number and tube length between HUVECs and hiPSC-derived ECs (Supplementary figure: Fig. S1E), suggesting functionally similarities between primary and hiPSC-derived ECs.

The differentiation of hiPSCs into ECs involved two stages: mesoderm induction and EC fate specification. Using quantitative real-time PCR (qRT-PCR), we observed a gradual increase in the gene expression levels of *PECAM1*, *CDH5*, *VWF*, and *BCL6B* during the hiPSC-EC differentiation process from D0 to D6. (Supplementary figure: Fig. S1F). The protein expression of VWF and VE-cadherin exhibited the same increasing pattern as their transcriptional levels throughout the hiPSC differentiation process. Surprisingly, the protein expression levels of BCL6B followed an opposite trend, becoming barely detectable between D5 and D6 of EC differentiation when EC markers peaked (Supplementary figure: Fig. S1G). This suggests that lower expression of BCL6B at the protein level is required for EC differentiation, whereas high expression of BCL6B at the protein level could affect EC generation.

### BCL6B overexpression suppressed EC differentiation from hiPSCs

**Fig. 1:**
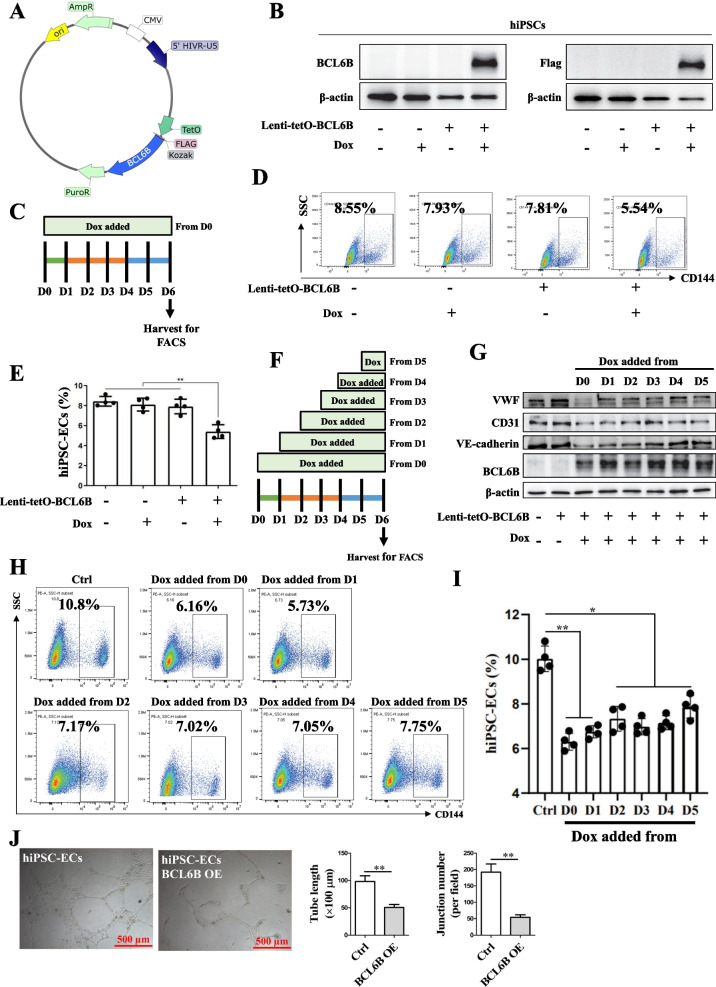
BCL6B overexpression suppressed ECs differentiation from hiPSCs. **A** Schematic illustration of the doxycycline-induced BCL6B overexpression vector using the tetO-on system. **B** Verification of doxycycline-induced BCL6B protein expression in the established inducible BCL6B-overexpression hiPSC line (BCL6B OE). **C** Schematic illustration of doxycycline addition to induce BCL6B overexpression. **D** Representative flow cytometry results of CD144^+^ cells in hiPSC-ECs from different groups. **E** Statistical analysis of the CD144^+^ cells in hiPSC-ECs from different groups. **, *P* < 0.01. **F** Schematic illustration of doxycycline addition at different time points. **G** The protein expression of VWF, CD31, VE-cadherin and BCL6B in D6 ECs derived from BCL6B OE hiPSC started to be treated with doxycycline at different time points. **H** Representative flow cytometry results of CD144^+^ cells in hiPSC-ECs from different groups. **I** Statistical analysis of the CD144^+^ cells in hiPSC-ECs from different groups. *, *P* < 0.05; **, *P* < 0.01. **J** Representative brightfield images of tube-like structures formed by hiPSC-ECs and BCL6B OE hiPSC-ECs (left panel), and quantitative analysis of tube length and junction number (right panel). OE, overexpression. **, *P* < 0.01

To investigate the involvement of BCL6B in EC differentiation, we developed a doxycycline inducible BCL6B-flag expression vector (Fig. 1A), and established an inducible hiPSC line to control the temporal expression of BCL6B upon the addition of doxycycline (Fig. 1B). Next, we treated the inducible hiPSC line with doxycycline to induce BCL6B overexpression throughout the EC differentiation process (Fig. 1C). The differentiation efficiency, assessed by flow cytometry analysis of CD144^+^ cells on D6, revealed that compared to the differentiation efficiency of control hiPSCs at 8.55% ± 0.13%, overexpression of BCL6B in hiPSCs resulted in a decrease in the differentiation of hiPSCs into ECs, with efficiencies of 5.54% ± 0.25%. In contrast, the group treated with doxycycline alone displayed no significant differences (Fig. 1D and E).

Given that the differentiation of EC from hiPSCs involves two stages and mesodermal cells express high levels of BCL6B (Supplementary figure: Fig. S1G), we further asked if EC differentiation efficiency could be temporally controlled by BCL6B activation. To test this, we treated the inducible hiPSCs with doxycycline at different time points until D6 of EC differentiation (Fig. 1F). Our western blot analysis confirmed that two days of doxycycline treatment were sufficient to achieve high expression levels of BCL6B (Fig. 1G). Consequently, we observed that overexpression of BCL6B significantly decreased the protein expression of VWF, VE-cadherin, and CD31 in hiPSC-ECs (Fig. 1G). Interestingly, regardless of the timing of doxycycline treatment, all groups exhibited decreased EC differentiation, although overexpression of BCL6B initiated in the early stages tended to show more severe inhibitory effects on EC differentiation (Fig. 1H and I). Furthermore, the overexpression of BCL6B during differentiation significantly attenuated the capacity of derived hiPSC-ECs to form a tubular structure, suggesting compromised EC function of BCL6B-overexpressing hiPSC-derives ECs (Fig. 1J). The above results clearly show that overexpression of BCL6B inhibits hiPSCs differentiation into ECs.

### Downregulation of BCL6B improved EC differentiation from hiPSCs

**Fig. 2:**
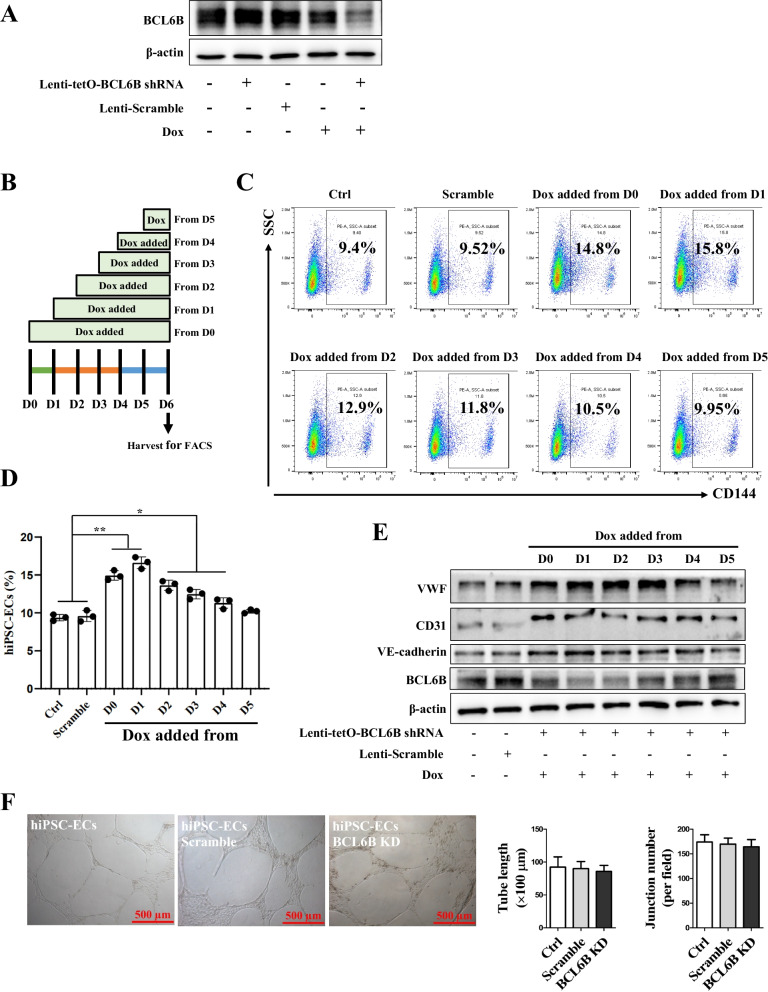
Downregulation of BCL6B improved ECs differentiation from hiPSCs. **A** Schematic illustration of the doxycycline-induced BCL6B shRNA vector using the tetO-on system. **B** Verification of doxycycline-induced BCL6B protein knock-down in the established inducible BCL6B-knock down hiPSC line (BCL6B KD). **C** Schematic illustration of doxycycline addition to induce BCL6B knockdown. **D** Representative flow cytometry results of CD144^+^ cells in hiPSC-ECs from different groups. **E** Statistical analysis of the CD144^+^ cells in hiPSC-ECs from different groups. *, *P* < 0.05; **, *P* < 0.01. **F** Protein levels of VWF, VE-cadherin, CD31, and BCL6B in D6 ECs derived from BCL6B KD hiPSC started to be treated with doxycycline at different time points. **G** Representative brightfield images of tube-like structures formed by hiPSC-EC and BCL6B KD hiPSC-ECs (left panel), and quantitative analysis of tube length and junction number (right panel)

To firmly establish a causal role for increased BCL6B activity in reduced EC differentiation efficiency and functional properties, we further investigated whether inhibiting the expression of BCL6B in hiPSCs would enhance their capacity to generate ECs. To achieve this, we constructed doxycycline-inducible vectors for effective silencing of endogenous BCL6B in hiPSCs upon doxycycline treatment (Fig. 2A). Subsequently, we initiated EC differentiation in the presence of doxycycline at six time points (D0, 1, 2, 3, 4, and 5) during the differentiation process (Fig. 2B). Our findings revealed that knockdown of BCL6B significantly increased EC differentiation, with efficiencies increasing from 9.4% ± 0.11% to 15.8% ± 0.23% (Fig. 2C and D). Furthermore, the highest differentiation efficiency was observed when BCL6B inhibition was initiated earlier than D1 of differentiation (Fig. 2C and D), which was consistent with the observation that a more profound inhibitory effect of BCL6B overexpression on EC differentiation was achieved at D0 of differentiation (Fig. 1I). Moreover, knocking down of BCL6B significantly increased the protein expression of VWF, VE-cadherin, and CD31 in hiPSC-ECs (Fig. 2E). However, the derived hiPSC-ECs with BCL6B knockdown did not demonstrate enhanced tubular structure formation capacity when compared to those without BCL6B inhibition during the differentiation process (Fig. 2F).

### BCL6B impaired blood vessel organoids generation

To further validate the critical role of BCL6B in vessel development within a more complex context, we generated vessel organoids (VOs) from hiPSCs. Briefly, hiPSCs were assembled into embryoid bodies (EBs) via centrifugation in ultra-low attachment 96-well plates, followed by a 24-h incubation prior to mesoderm induction using CHIR99021 and BMP4 (Supplementary figure: Fig. S2A). After 3 days, the differentiated EBs were treated with VEGF and forsklin for 2 additional days for further vascular lineage development. Subsequently, the EBs were embedded in Matrigel:Collagen I (1:1) gels to promote EC sprouting and structure maturation. Brightfield imaging of VOs showed a significant increase in the size of VOs throughout the differentiation process (Supplementary figure: Fig. S2B). Confocal imaging revealed the formation of complex, interconnected vascular networks by CD31^+^ ECs (Supplementary figure: Fig. S2C). Moreover, these self-organizing 3D VOs exhibited proper localization of pericytes, defined by the molecular markers ⍺-SMA and PDGFRβ. Additionally, the vessel-like structures were enveloped by a basement membrane and mesenchymal fibroblasts, as confirmed by immunostaining for Collagen IV (Col IV) and Vimentin, respectively (Supplementary figure: Fig. S2D).

We further performed qRT-PCR to determine the expression of pluripotency and vascular-specific marker genes in VOs. At D15 of differentiation, hiPSC-VOs were negligible for *OCT4*, *NANOG*, and *SOX2*, and showed drastic upregulation of EC markers (*PECAM1*, *CDH5*, *VWF*, *and VEGFR2*) and a pericyte marker (*PDGFRβ*) (Supplementary figure: Fig. S2E). In line with this, our flow cytometry results revealed that approximately 33.4% of total cell population in the hiPSC-VOs were CD144^+^ ECs at D15 of differentiation (Supplementary figure: Fig. S2F). To determine whether hiPSC-VOs respond to proinflammatory cytokines with a pro-adhesive phenotype, we challenged the hiPSC-VOs with lipopolysaccharides (LPS). Immunofluorescence analysis showed a significant increase in intracellular adhesion molecule-1 (ICAM1) level upon LPS treatment (Supplementary figure: Fig. S2G). Thus, we have successfully established a fully structured and functional human VO model.

**Fig. 3:**
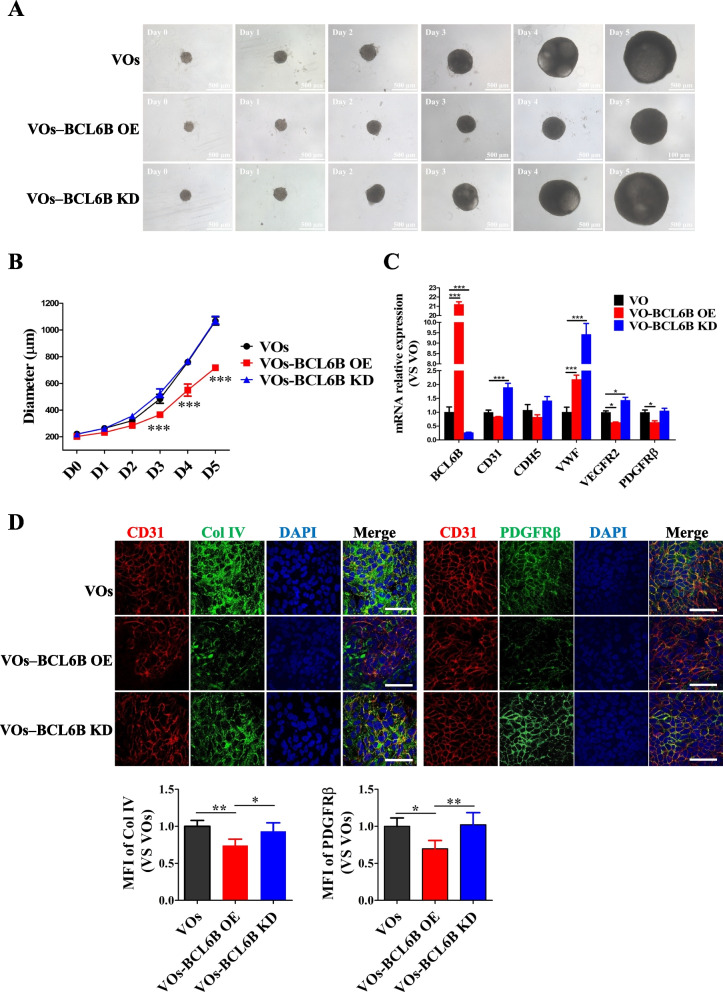
Overexpression of BCL6B impaired VOs development. **A** Brightfield images illustrating the development of VOs from hiPSCs with or without BCL6B gene manipulation over a 6-day differentiation period. **B** Statistical analysis of the diameter of VOs derived from control, BCL6B OE, and BCL6B KD hiPSCs. N = 16; ***, *P* < 0.001. **C** mRNA expression of vessel markers (*VWF*, *CDH5*, *CD31*, *VEGFR2* and *PDGFRβ*) and *BCL6B* in D15 VOs (n = 10). Data normalized to *GAPDH* expression. **D** Immunostaining and quantification of PDGFRβ and Col IV in D15 VOs. Col IV, Collagen IV. *, *P* < 0.05; **, *P* < 0.01

With the successful establishment of blood VOs, we proceeded to generate BCL6B gene-perturbed blood VOs using the previously developed inducible hiPSC lines. Remarkably, the control VOs showed a gradual increase in the size from 219.9 ± 3.5 µm at D0 to 1067.3 ± 24.6 µm at D5 (Fig. 3A). In contrast, BCL6B overexpression, particularly after D2 of differentiation, significantly repressed the growth of VOs (from 212.6 ± 3.8 µm at D0 to 717.3 ± 24.2 µm at D5), whereas BCL6B knockdown had little effect on the size of VOs (from 220.9 ± 4.5 µm at D0 to 1073.4 ± 29.2 µm at D5) (Fig. 3A and B). BCL6B knockdown significantly upregulated the expression levels of *CD31*, *VWF* and *VEGFR2*, while BCL6B overexpression downregulated the expression levels of *VEGFR2* and *PDGFRβ* in D15 hiPSC-VOs (Fig. 3C). Importantly, Immunofluorescence staining showed that both Col IV and PDGFRβ proteins were significantly downregulated upon BCL6B overexpression in day 15 hiPSC-VOs (Fig. 3D). Collectively, these data strongly suggest that BCL6B is a negative regulator in EC differentiation and vascular network formation in hiPSC-derived VOs.

### BCL6B inhibited EC differentiation from hiPSCs by repressing ETV2 transcription

**Fig. 4:**
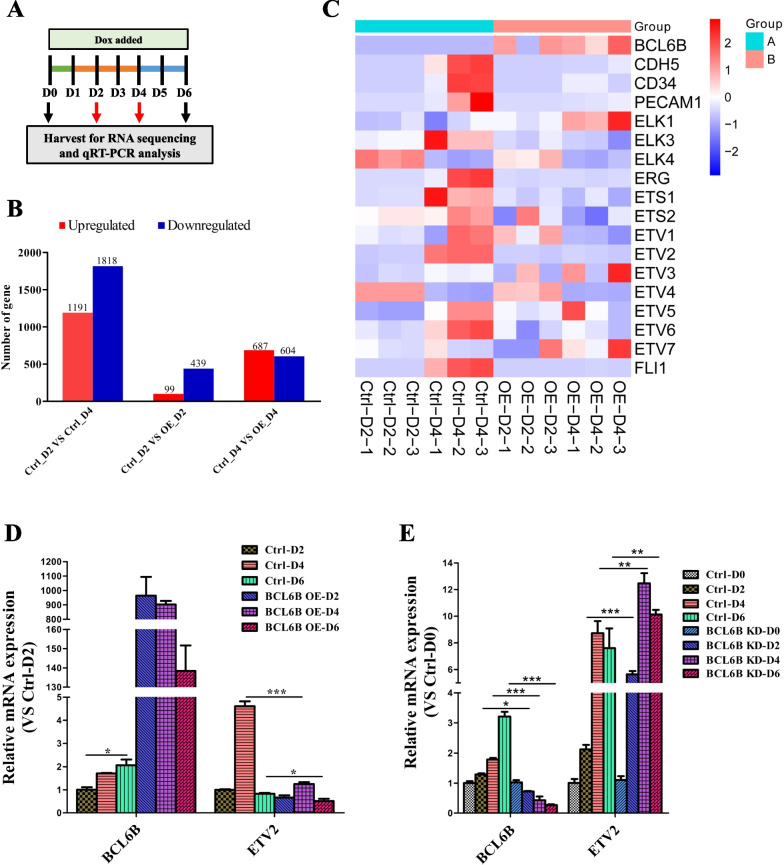
RNA-seq analysis showing ETV2 inhibition by BCL6B during EC differentiation from hiPSCs. **A** Schematic illustration of doxycycline addition during EC differentiation and sample collection for analysis. **B** Bar graphs show upregulated and downregulated gene numbers in control (Ctrl) and BCL6B overexpression (OE) differentiating hiPSCs at day 2 and 4 respectively. **C** Heatmap showing expression levels of the ETS-domain transcription factors in differentiating hiPSCs with indicated treatments. **D** mRNA expression of *BCL6B* and *ETV2* during EC differentiation from control or BCL6B OE hiPSCs upon doxycycline treatment as shown in **A**. Data normalized to *GAPDH* expression. *, *P* < 0.05; ***, *P* < 0.001. **E** mRNA expression of *BCL6B* and *ETV2* during EC differentiation from control or BCL6B KD hiPSCs upon doxycycline treatment as illustrated in **A**. Data normalized to *GAPDH* expression. *, *P* < 0.05; **, *P* < 0.01; ***, *P* < 0.001

To identify the potential downstream targets regulated by BCL6B during EC differentiation, we conducted RNA-seq analysis in control (Ctrl) and BCL6B overexpression (OE) differentiating hiPSCs at different differentiation stages (day 2 and 4) (Fig. 4A). RNA sequencing analysis showed that compared to day 2 differentiating hiPSCs, there were 1191 up-regulated genes and 1818 down-regulated genes, respectively, in day 4 differentiating hiPSCs (Fig. 4B). Gene Ontology (GO) enrichment analysis of differently expressed genes (DEGs) between day 4 and day 2 differentiating hiPSCs revealed significant enrichments of multiple cell/tissue/system developmental processes and cell differentiation (Supplementary figure: Fig. S3A), further confirming that our differentiation protocol effectively and specifically drives vascular development and EC differentiation. Moreover, KEGG (Kyoto Encyclopedia of Genes and Genomes) pathway enrichment analysis showed multiple important cellular development and differentiation signaling pathways (Rap1, Hippo, stem cell pluripotency, Wnt, and Calcium) were activated during EC differentiation from hiPSCs (Supplementary figure: Fig. S3B). As expected, hundreds of genes were modulated by BCL6B overexpression in differentiating hiPSCs, peaking at day 4 (Fig. 4B), and importantly, a variety of cell/tissue/system developmental and differentiation processes (circulatory system and tube development) as well as multiple KEGG pathways (MAPK, Rap1, Ras, Calcium, Relaxin, Wnt, PI3K-Akt, cAMP, and stem cell pluripotency) were dramatically modified by BCL6B overexpression (Supplementary figure: Fig. S3C-3F), supporting an important regulatory role for BCL6B in EC differentiation.

Several transcription factors, particularly the ETS-domain transcription factors, have critical roles in vascular EC differentiation or vascular development [26, 27]. These factors include ETS proto-oncogene 1 (ETS1), ETS proto-oncogene 2 (ETS2), ETS transcription factor ERG, ETS transcription factor ELK3, Fil1, ETS variant transcription factor 2 (ETV2) [18, 28] and ETS variant transcription factor 6 (ETV6). It therefore plausible for us to speculate that BCL6B mediates EC differentiation and vascular development by regulating ETS-domain transcription factors. Indeed, heatmap showed that while majority of ETS-domain transcription factors (ELK3, ERG, ETS1, ETS2, ETV1, ETV2, ETV5, ETV6, and FLI1) were significantly increased during EC differentiation from hiPSCs, two of them (ELK4 and ETV4) were decreased, with the most significant changes at day 4 (Fig. 4C). Crucially, we observed an opposite trend for most of these genes upon BCL6B overexpression (Fig. 4C), inferring a negative regulatory role for BCL6B in these genes during EC differentiation from hiPSCs. Then we performed qRT-PCR analysis to confirm above observation. We verified that the induction of BCL6B overexpression in hiPSCs through doxycycline significantly decreased the mRNA expression of ETV2 during the EC specification stage (Fig. 4D), which was consistent with the result of RNA-seq. Previous study showed that ETV2 activation facilitates EC differentiation from hiPSCs [29]. We found that the abrogation of BCL6B expression significantly enhanced the transcription of ETV2 during EC differentiation (Fig. 4E).

**Fig. 5:**
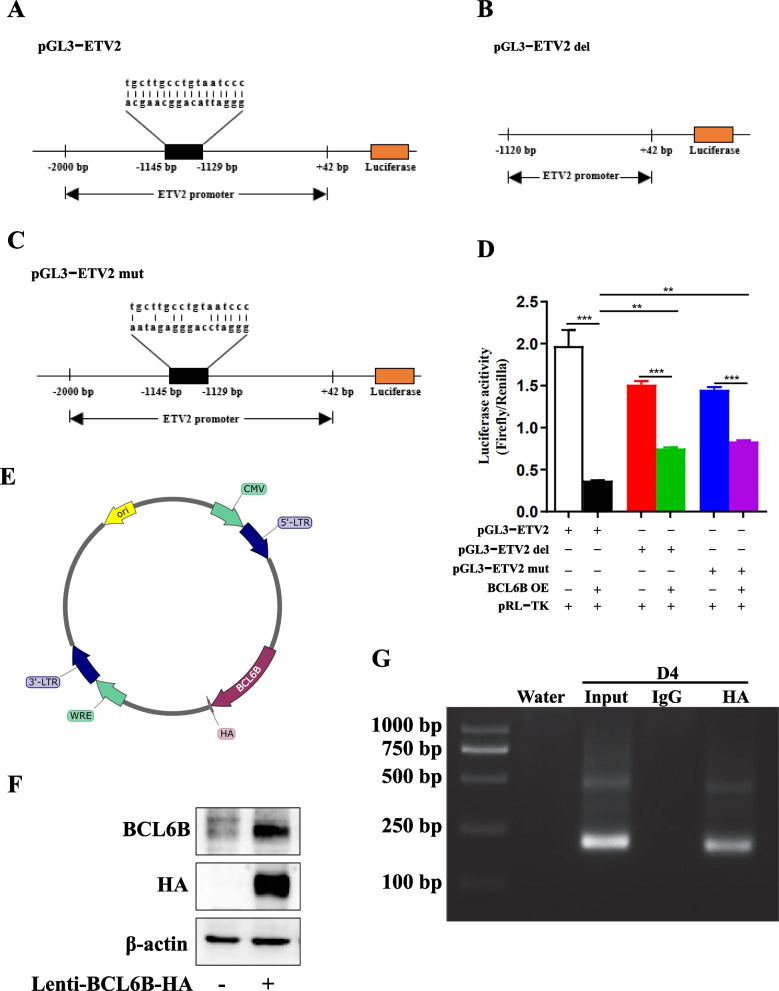
BCL6B transcriptionally inhibited ETV2 expression. **A**–**C** Schematic illustration of three luciferase reporter vectors carrying the full-length (**A**), truncated (**B**) or mutated (**C**) ETV2 gene promoters. **D** Luciferase activity in 293 T cells after transfection with luciferase reporter vectors, with or without the BCL6B overexpression vector for 24 h. The renilla luciferase vector (pRL-TK) was co-transfected as a control. **, *P* < 0.01; ***, *P* < 0.001. **E** Schematic illustration of BCL6B with HA-tag overexpression vector. **F** Verification of BCL6B protein expression in hiPSCs as determined by western blot. **G** ChIP-PCR assay was performed to determine the binding of BCL6B to ETV2 promoter

It has been well-known that BCL6B is a transcription repressor. To further investigate whether BCL6B is capable of binding to ETV2 promoter and repress its transcription, we first analyzed the ETV2 promoter using JASPAR website (https://jaspar.elixir.no/), and found that a putative binding site of BCL6B upstream of the ETV2 promoter region between − 1145 bp and − 1129 bp. We then generated three different ETV2 promoter-luciferase constructs, one carrying a 2-kb ETV2 promoter (pGL3-ETV2, Fig. 5A), another one carrying a 1.1-kb truncated promoter by deleting the BCL6B binding site (pGL3-ETV2-del, Fig. 5B), and the third one carrying a 2.0 kb promoter but in which the BCL6B binding site is mutated (pGL3-ETV2-mut, Fig. 5C). We next examined the effect of BCL6B on the ETV2 luciferase reporter in 293 T cells, and found that BCL6B overexpression dramatically decreased the full-length ETV2 gene promoter activity. In response to BCL6B overexpression, both constructs carrying either a truncated (pGL3-ETV2-del) or a mutated (pGL3-ETV2-mut) ETV2 gene promoter exhibited a much higher level of promoter activity, compared to pGL3-ETV2 (Fig. 5D). Importantly, a much lower ETV2 gene promoter activity was observed in both the truncated (pGL3-ETV2-del) and the mutated (pGL3-ETV2-mut) ETV2 gene promoter vector in absence of BCL6B overexpression, suggesting that BCL6B binding element within ETV2 gene promoter is important for ETV2 gene transcription at base level. To further confirm the binding of BCL6B to ETV2 promoter, we constructed BCL6B overexpressing vector carrying a HA-tag and infected differentiating hiPSCs with this vector for 4 days, followed by ChIP-PCR analysis using an anti-HA antibody (Fig. 5E and F). The expected size (190 bp) of ETV2 promoter amplicon containing the BCL6B binding element was detected in the formaldehyde-mediated DNA–protein complexes immunoprecipitated with anti-HA antibody (Fig. 5G). On the other hand, no signal was detected when normal rabbit IgG was added (Fig. 5G). Our results show that BCL6B is able to bind to ETV2 promoter and suppresses ETV2 gene expression.

**Fig. 6:**
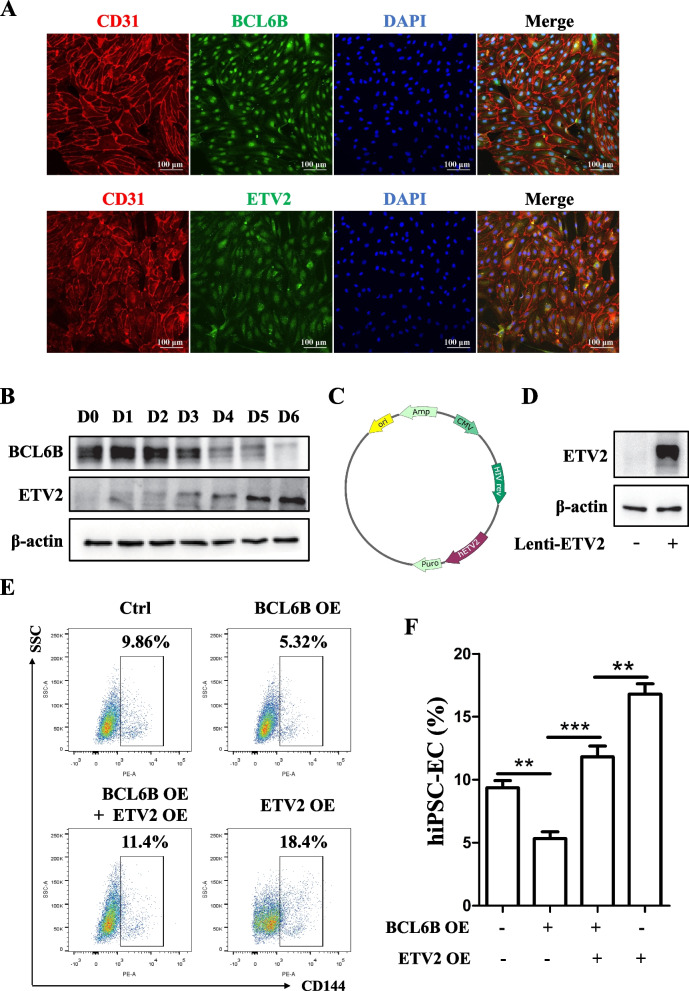
The inhibitory effect of BCL6B overexpression on EC differentiation was rescued by ETV2 overexpression. **A** Immunostaining of BCL6B and ETV2 in hiPSC-derived ECs. **B** Protein expression levels of BCL6B and ETV2 throughout EC differentiation from D0 to D6, as determined by western blot. **C** Schematic illustration of ETV2 overexpression vector. **D** Verification of ETV2 protein expression in hiPSCs as determined by western blot. **E** Representative flow cytometry results of CD144^+^ cells in hiPSC-ECs from different groups. **F** Statistical analysis of the CD144^+^ cells in hiPSC-ECs from different groups. **, *P* < 0.01; ***, *P* < 0.001

Next, to further identify the role of BCL6B-ETV2 signal axis in EC differentiation from hiPSCs, we firstly conducted immunofluorescence staining of BCL6B and ETV2 in hiPSC-derived ECs. The result showed both BCL6B and ETV2 were mainly expressed in nuclei in hiPSC-derived ECs (Fig. 6A) and HUVECs (Supplementary figure: Fig. S4A). To further confirm the co-expression of BCL6B and ETV2 in hiPSC-derived ECs, we generated a lentiviral vector with BCL6B overexpression along with EGFP and transfected it into hiPSCs, followed by EC differentiation (Supplementary figure: Fig. S4B). EGFP^+^ differentiated ECs (BCL6B^+^/CD144^+^) were sorted from differentiating hiPSCs at day 6 (Supplementary figure: Fig. S4C) and subjected to qRT-PCR analysis (Supplementary figure: Fig. S4D) and immunofluorescence staining assay (Supplementary figure: Fig. S4E), respectively. Data from qRT-PCR analysis showed that ETV2 gene expression was significantly downregulated upon BCL6B overexpression in sorted EGFP^+^ differentiated ECs (Supplementary figure: Fig. S4, C and D). Immunofluorescence staining also confirmed co-expression of BCL6B and ETV2 in hiPSC-derived ECs (Supplementary figure: Fig. S4E).

Additionally, while BCL6B expression was gradually decreased during EC differentiation from hiPSCs, we observed a gradual increasing protein expression of ETV2 during same period (Fig. 6B). Subsequently, we constructed lentiviral vector with ETV2 overexpression (Fig. 6C, D), and conducted a co-gene transduction during EC differentiation from hiPSCs. As expected, while BCL6B and ETV2 overexpression alone significantly reduced and increased EC differentiation from hiPSCs, the inhibitory effect of BCL6B overexpression on EC differentiation was largely rescued by ETV2 overexpression (Fig. 6E, F). Collectively, these data strongly suggest that BCL6B suppresses EC differentiation through inhibiting ETV2.

## Discussion

ECs derived from hiPSCs with their unlimited expansion potential, are considered an alternative cell source for experimental studies, and especially, therapeutic approaches [30]. Generally, there are 3 primary methods for EC derived from differentiating embryonic stem cells (ESCs) or iPSCs [31]. First, ESC and iPSC can be differentiated into 3D EBs, in which spontaneously undergo differentiation into the three germ layers: ectoderm, endoderm, and mesoderm, with emergence of numerous lineages of cells. Addition of a variety of growth factors promotes EC differentiation within the EB. In a second method, coculture of differentiating ESC or iPSC with other stromal cells to promote EC lineage differentiation from the mesoderm. Finally, EC differentiation performed in 2D culture on cell culture plates coated with Matrigel or other proteins using specific culture mediums with sequentially added recombinant growth factors. Several independent laboratories have successfully generated ECs from hiPSCs or human pluripotent stem cells (hPSCs). Amy Cochrane. et al. [32] reported that the efficiency of EC differentiation from hiPSCs was 62%. An approximately 15% CD31 + cells was observed in differentiating EBs derived from mouse ESCs [33]. Moreover, about 18–33% differentiation efficiency was achieved with some hiPSC lines [34–36]. In our study using the adapted protocol, we observed a basal differentiation efficiency of approximately 10%, which was much lower than that (61.8–88%) in original protocol [22], while Yingyi Quan et al. [37] reported a 43.5% differentiation efficiency in their study when followed the original protocol. Although defined conditions have been described, a very low efficiencies of EC differentiation (< 1.5%) was also observed in the early protocols [38]. Multiple factors, for example, fetal bovine serum (FBS), bovine serum albumin (BSA), the quality and source of stem cell lines, and growth factors, may attribute to the different EC differentiation efficiency reported in various studies.

BCL6B is reported to be involved in a diversity of biological functions, including tumor suppression[4, 6, 8], immune response[3], stem cell self-renew[2], and vascular angiogenesis[10], all of which are related to its function as a transcriptional repressor. Interestingly, it has been reported that BCL6B-deficient mice are viable and no gross anatomical abnormalities were observed after birth, with altered T cell activation [39] and hematopoiesis [40]. However, later studies showed that BCL6B gene disruption in vivo markedly impaired angiogenesis in the mouse retina and wounded skin [10], as well as choroidal neovascularization lesions and ocular edema in the neuroretina [9], indicating an essential role for BCL6B in ocular neovascularization and edema. These observations suggest that while BCL6B is critical for neovascularization under pathological conditions, it may be redundant during embryonic development and under physiological conditions due to gene duplication or complementary effects from other paralogous genes.

Given its role as a transcriptional repressor, we anticipated a broader functional impact with BCL6B. Online database indicated a high mRNA expression level of BCL6B in human ECs [10], which is consistent with our qRT-PCR data. However, at the protein level, the BCL6B expression is notably low. This discrepancy adds intrigue to unveiling its role in EC development and function. We constructed inducible overexpression and knockdown vectors to manipulate BCL6B expression during EC differentiation from hiPSCs and VO generation from hiPSCs. The data clearly demonstrated that BCL6B inhibits EC differentiation. Although we clearly observed that knockdown of BCL6B in hiPSCs significantly increased EC differentiation, impaired BCL6B expression in hiPSC-derived ECs exhibited negligible effect on tube formation. However, Ohnuki et al. reported that BCL6B knockdown abrogated EC network formation in HUVECs [10], indicating that BCL6B may play a distinct role in primary and hiPSC-derived ECs. Similar to tube formation, we also found that BCL6B overexpression impaired the size of VOs during the first 5 days of differentiation, whereas knockdown of BCL6B had little effect on VO size.

Mechanistically, we uncovered that BCL6B inhibits ETV2 transcription, consequently inhibiting EC differentiation. It has been widely known that ETV2 is a master transcriptional regulator required for the development of both endothelial and erythropoietic lineages [41]. Of note, a recent study [42] has shown a differential ETV2 threshold requirement for endothelial and erythropoietic development. Specifically, a low-to-moderate level of ETV2 expression is sufficient to induce and sustain EC differentiation, while a higher level of ETV2 expression is required for erythropoiesis rather than for vasculogenesis. Therefore, this interesting finding together with our data indicate that fine-tuning regulation of ETV2 gene expression by BCL6B is essential for EC differentiation from hiPSCs or maintaining EC functions. Moreover, as a transcriptional repressor, BCL6B is expected to have numerous binding sites in the genome, and other potential targets of BCL6B may contribute to the inhibition of EC differentiation from hiPSCs. A comprehensive approach to interrogate the potential targets of BCL6B would involve using ChIP-seq technology. Additionally, the inconsistency between BCL6B mRNA and protein expression during EC differentiation suggests a negative-feedback regulation in BCL6B gene regulation during EC differentiation from hiPSCs, which merit further investigation.

ECs line the interior of all blood and lymphatic vessels, playing key roles in delivering oxygen and nutrients, regulating blood flow, modulating immune cell trafficking and maintaining tissue homeostasis [43]. Vascular EC dysfunction is central to the progression of most chronic conditions, including ischemic heart disease [44] and stroke [45], the top two global causes of mortality. A published study has demonstrated that BCL6B contributes to ocular vascular diseases via notch signal silencing [9].

## Conclusion

In conclusion, this study is the first time to demonstrate that BCL6B can modulate EC differentiation as well as blood VO development, and that BCL6B exerts its modulatory effect on EC differentiation programmed by fine-tuning regulation of ETV2 gene transcription. Our study reveals a new role of BCL6B in EC development and function, making it a potential target for precision therapy of vascular diseases.

### Supplementary Information

**Supplementary Material 1.**

**Supplementary Material 2.**

## Data Availability

All data generated or analyzed during this study are included in this published article. Data generated during the current study are available from the corresponding author on reasonable request.

## Abbreviations

BCL6B: B-cell CLL/lymphoma 6 member B
EC: Endothelial cell
ETV2: ETS variant transcription factor 2
hiPSC: Human induced pluripotent stem cell
VO: Vessel organoids
qRT-PCR: Quantitative real-time PCR
BCL6: B-cell lymphoma 6
BTB/POZ: Broad-Complex, Tramtrack and Bric a brac/POxvirus and Zinc finger domain
HUVECs: Human umbilical vein endothelial cells
EBs: Embryoid bodies
Col IV: Collagen IV
LPS: Lipopolysaccharides
ICAM1: Intracellular adhesion molecule-1
ETS1: ETS proto-oncogene 1
ETS2: ETS proto-oncogene 2
ETV6: ETS variant transcription factor 6
ESCs: Embryonic stem cells
hPSCs: Human pluripotent stem cells
FBS: Fetal bovine serum
BSA: Bovine serum albumin
RNA-Seq: RNA sequencing
ChIP: Chromatin immunoprecipitation

## Declarations

### Artificial intelligence

The authors declare that artificial intelligence is not used in this study.
