# Supplementary material for: BCL6B-dependent suppression of ETV2 hampers endothelial cell differentiation
Source: Stem Cell Res Ther. 2024 Jul 29;15:226. doi: 10.1186/s13287-024-03832-y (PMC11287929; doi:10.1186/s13287-024-03832-y)
Supplement: Supplementary file 2 — Supplementary Material 2. [file 13287_2024_3832_MOESM2_ESM.pdf]

Supplemental information

BCL6B-dependent Suppression of ETV2 Hampers Endothelial Cell Differentiation

Zhonghao Li, Wei Wu, Qiushi Li, Xin Heng, Wei Zhang, Yinghong Zhu, Lin Chen, Ziqi Chen, Mengcheng Shen, Ning Ma, Qingzhong Xiao, Yi Yan

Figure S1. Generation and characterization of hiPSC-derived ECs

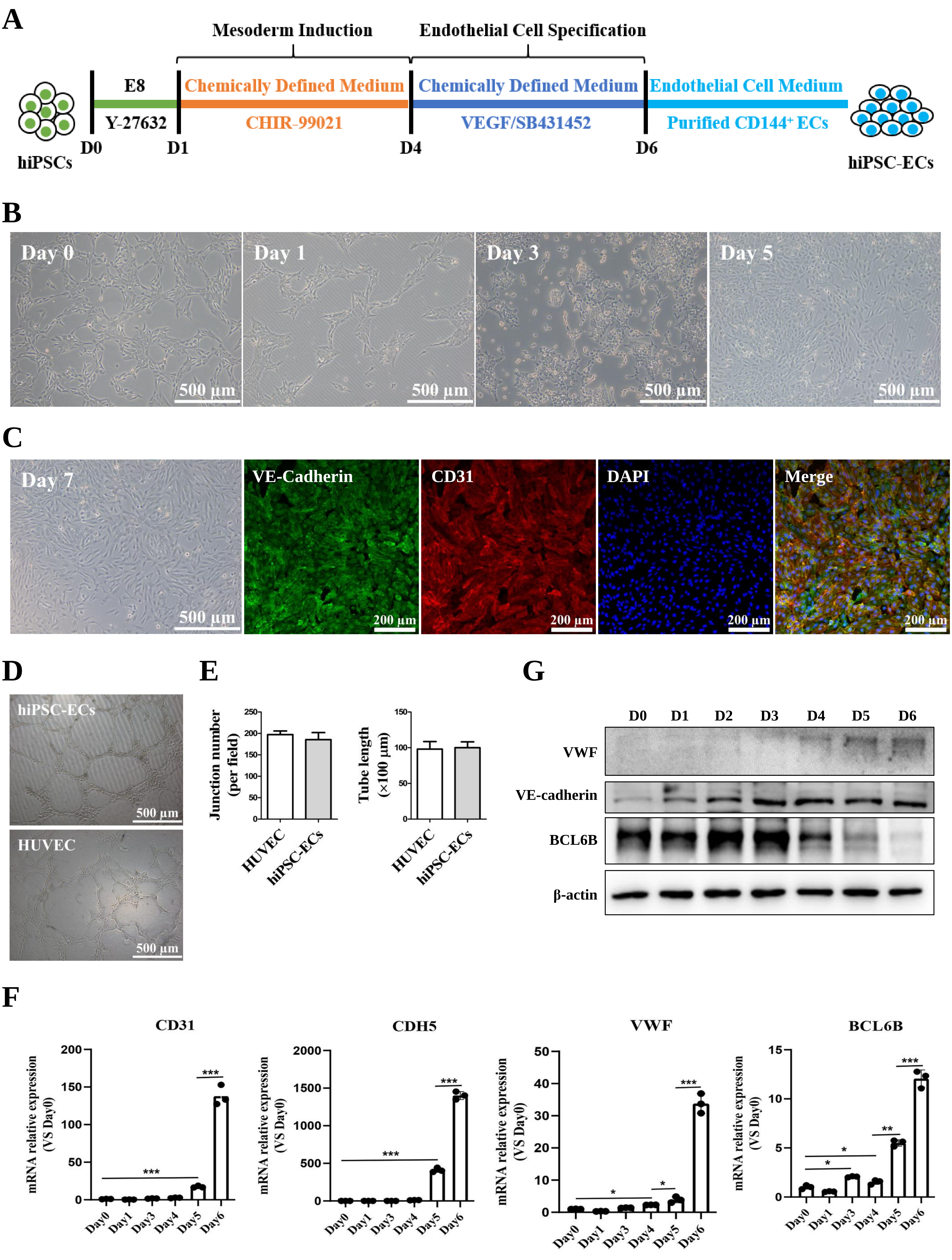

Figure S1. Generation and characterization of hiPSC-derived ECs

(A) Schematic illustration outlining the strategy for EC differentiation from hiPSCs. The differentiation of hiPSCs into ECs involved two stages: mesoderm induction and EC specification, with the entire process typically taking 6 days.

(B) Brightfield images depicting the cell morphology at different time points during EC differentiation.

(C)Brightfield image showing purified ECs at D7, along with confocal immunofluorescence images highlighting EC markers VE-Cadherin (green) and CD31 (red) with DAPI (blue).

(D) Brightfield images displaying tube-like structures formed by hiPSC-ECs and HUVEC.

(E) Quantitative analysis of junction number and tube length.

(F) mRNA expression of EC markers (*VWF*, *CDH5*, *CD31*) and *BCL6B* during EC differentiation.

(G) Protein levels of VWF, VE-Cadherin and BCL6B throughout EC differentiation from D0 to D6, as determined by western blot.

Figure S2. Generation and characterization of blood VOs

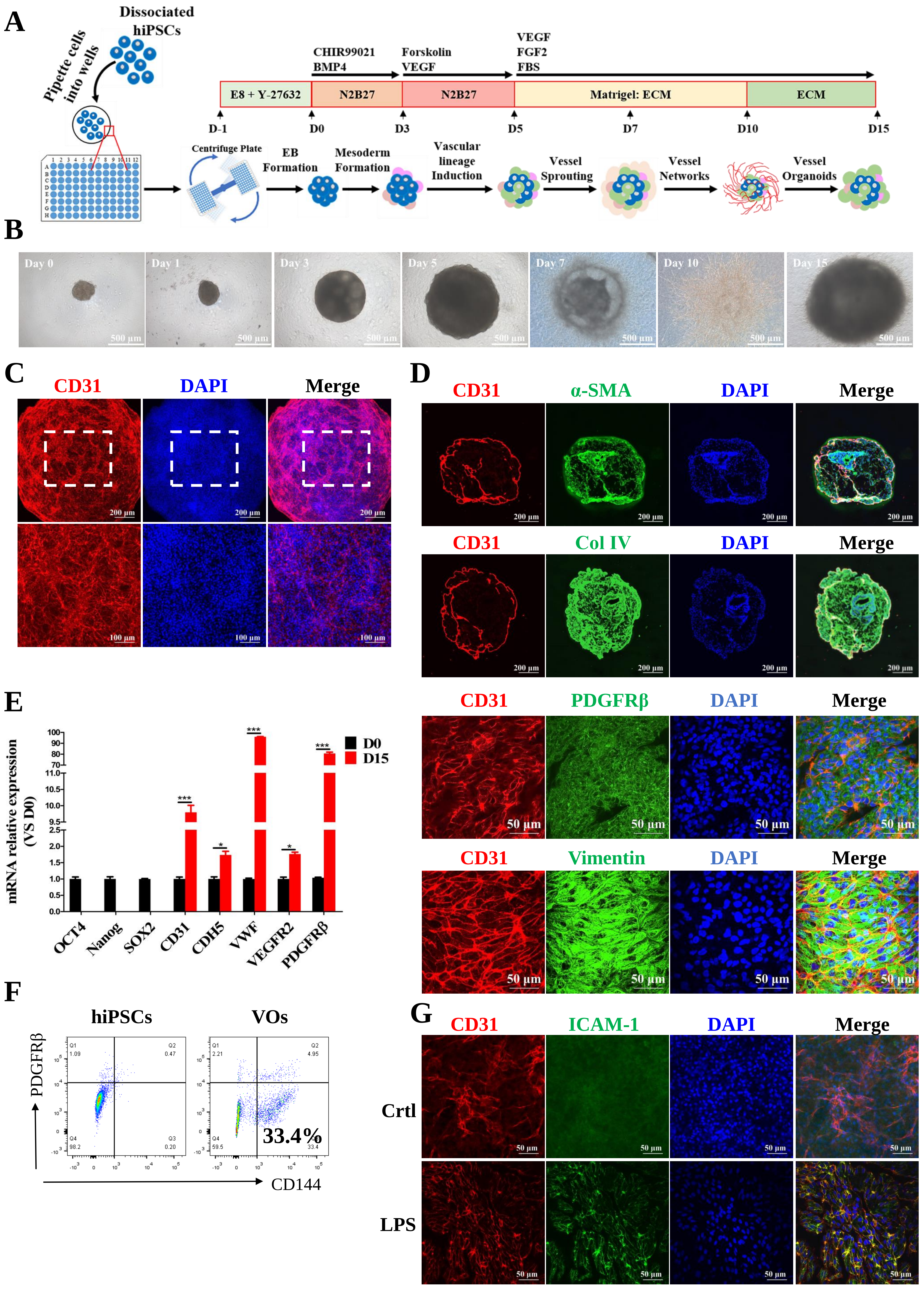

Figure S2. Generation and characterization of blood VOs

(A) A schematic diagram illustrating the protocol used to generate VOs from hiPSCs.

(B) Brightfield images depicting the development of VOs over a 15-day differentiation period.

(C) Confocal imaging of CD31 in D15 VOs.

(D) Immunostaining of α-SMA, Col IV and CD31 in D15 VOs. α-SMA, alpha- smooth muscle cell actin; Col IV, Collagen IV.

(E) mRNA expression of stem markers (*Nanog*, *OCT4*, *SOX2*) and vessel markers (*VWF*, *CDH5*, *CD31*, *VEGFR2* and *PDGFRβ*) in D15 VOs (n=10) compared to D0. Data normalized to *GAPDH* expression.

(F) Flow cytometry analysis of $PDGFR\beta^{+}$ and $CD144^{+}$ cells in D15 VOs.

(G) Immunostaining of ICAM-1 expression in D15 VOs after treatment of LPS (2 µg/mL) for 24 hours.

Figure S3. RNA-Seq analysis showing GO enrichment and pathway changed during EC differentiation with or without BCL6B overexpression

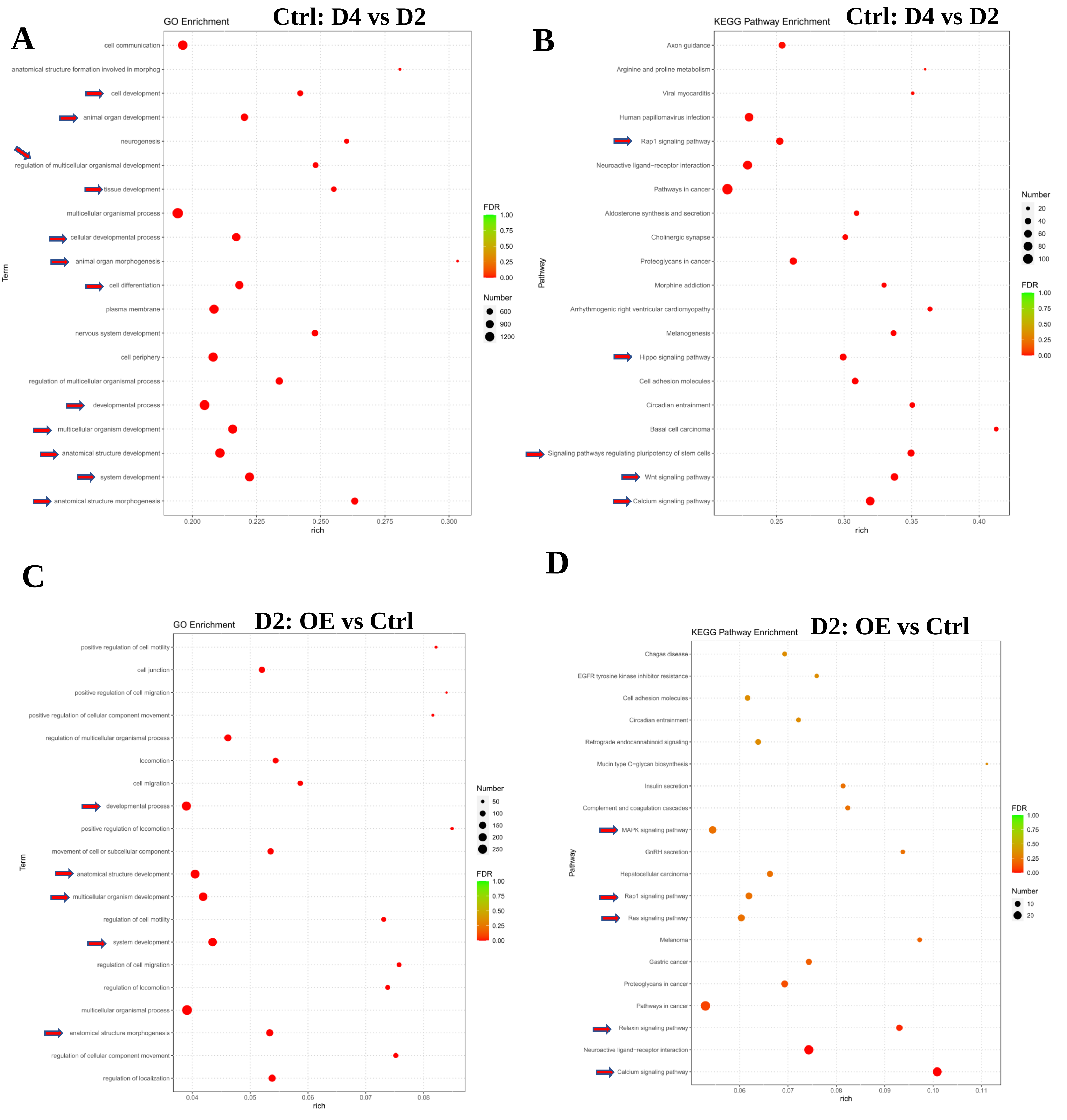

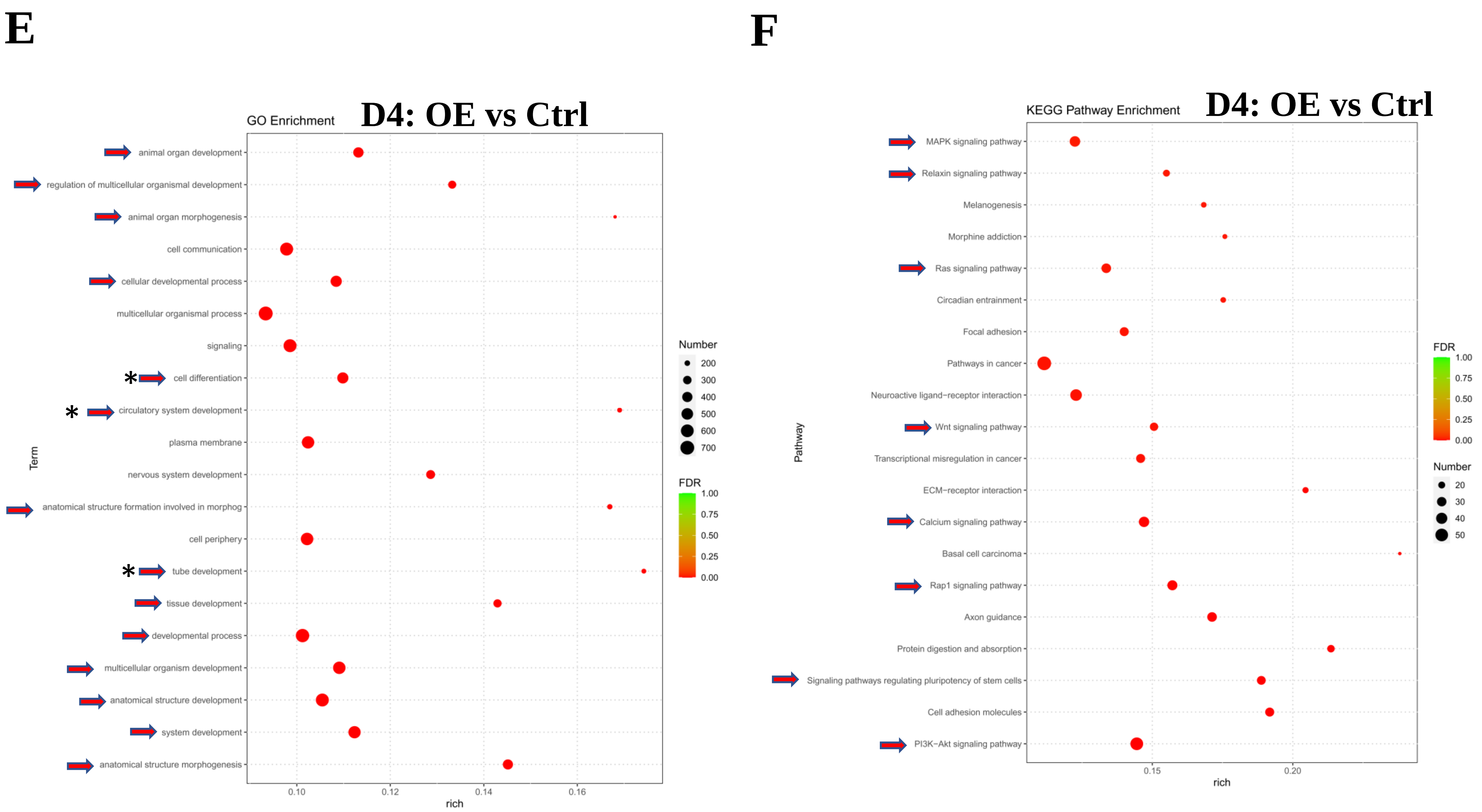

E
GO Enrichment
D4: OE vs Ctrl
animal organ development
regulation of multicellular organismal development
animal organ morphogenesis
cell communication
cellular developmental process
multicellular organismal process
signaling
* cell differentiation
* circulatory system development
plasma membrane
nervous system development
anatomical structure formation involved in morphog
cell periphery
* tube development
tissue development
developmental process
multicellular organism development
anatomical structure development
system development
anatomical structure morphogenesis
Term
rich
0.10
0.12
0.14
0.16
Number
200
300
400
500
600
700
FDR
1.00
0.75
0.50
0.25
0.00
F
KEGG Pathway Enrichment
D4: OE vs Ctrl
MAPK signaling pathway
Relaxin signaling pathway
Melanogenesis
Morphine addiction
Ras signaling pathway
Circadian entrainment
Focal adhesion
Pathways in cancer
Neuroactive ligand−receptor interaction
Wnt signaling pathway
Transcriptional misregulation in cancer
ECM−receptor interaction
Calcium signaling pathway
Basal cell carcinoma
Rap1 signaling pathway
Axon guidance
Protein digestion and absorption
Signaling pathways regulating pluripotency of stem cells
Cell adhesion molecules
PI3K−Akt signaling pathway
Pathway
rich
0.15
0.20
FDR
1.00
0.75
0.50
0.25
0.00
Number
20
30
40
50

Figure S3. RNA-Seq analysis showing GO enrichment and pathway changed during EC differentiation with or without BCL6B overexpression

(A) GO enrichment analysis of differently expressed genes (DEGs) between day 4 and day 2 differentiating hiPSCs.

(B) KEGG pathways of DEGs between day 4 and day 2 differentiating hiPSCs.

(C) GO enrichment analysis of DEGs at day 2 of differentiating hiPSCs between control (Ctrl) and BCL6B overexpression (OE).

(D) KEGG pathways of DEGs at day 2 of differentiating hiPSCs between control (Ctrl) and BCL6B overexpression (OE).

(E) GO enrichment analysis of DEGs at day 4 of differentiating hiPSCs between control (Ctrl) and BCL6B overexpression (OE).

(F) KEGG pathways of DEGs at day 4 of differentiating hiPSCs between Ctrl and OE.

Figure S4. BCL6B inhibited ETV2 expression

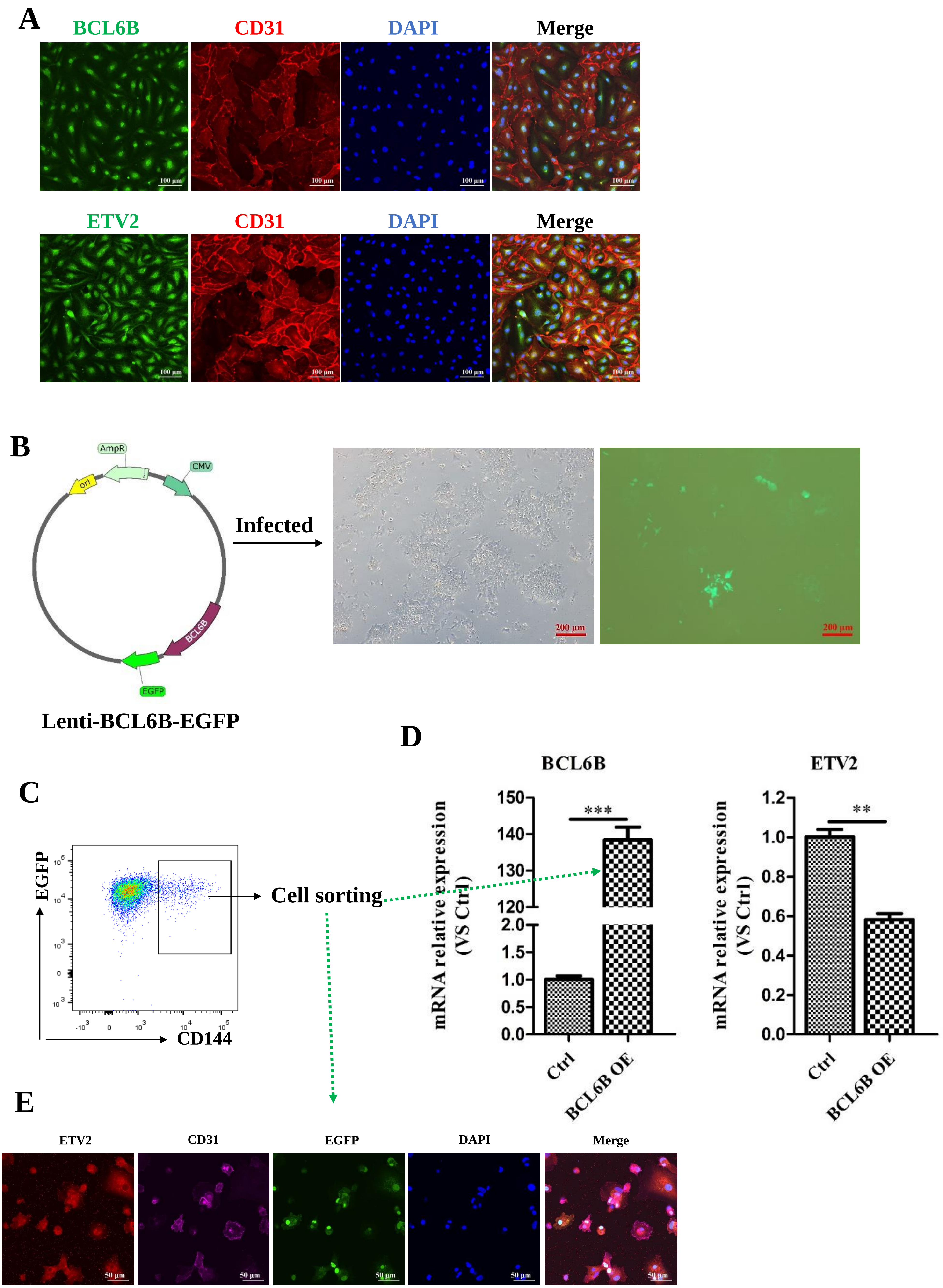

Figure S4. BCL6B inhibited ETV2 expression

(A) Immunofluorescence staining of BCL6B and ETV2 in HUVEC.

(B) Schematic illustration of BCL6B with EGFP overexpression vector and transfected into hiPSCs.

(C) Cell sorting of EGFP+ differentiated ECs at day 6.

(D) mRNA expression of BCL6B and ETV2 expression in control and sorted ECs ($EGFP^+/CD144^+$).

(E) Immunofluorescence staining of CD31 and ETV2 in sorted ECs ($EGFP^+/CD144^+$).
